# Immediate Massive Posttraumatic Pseudolipoma of the Buttocks: A Case of a Heterotopic “Love Handle”

**DOI:** 10.1097/GOX.0000000000001887

**Published:** 2018-09-06

**Authors:** Jocelyn C. Zajac, Max Mandelbaum, James M. Economides, Jerry W. Chao

**Affiliations:** From the * Department of Surgery, Division of Plastic and Reconstructive Surgery, The George Washington University Hospital, Washington, D.C.; † Department of Plastic and Reconstructive Surgery, MedStar Georgetown University Hospital, Washington, D.C.

Sir,

Posttraumatic pseudolipoma refers to an unencapsulated fatty mass presenting after blunt trauma.^1^ A leading hypothesis suggests their etiology as adipose tissue herniated through traumatic defects in the superficial fascial system or surrounding septa.^1–3^ Others postulate that trauma and hematoma formation triggers inflammatory cascades that upregulate fat precursor cells to differentiate into mature adipocytes. They generally occur in areas of high adiposity such as the hip, thigh, or gluteal regions and have a 12-to-1 predilection for females.^4,5^ The vast majority of pseudolipomas described in the existing literature note a delayed formation occurring months to years following trauma, with most developing after a mean of 2 years and reaching a modest size of under 10 cm.^2^

**Fig. 1.**
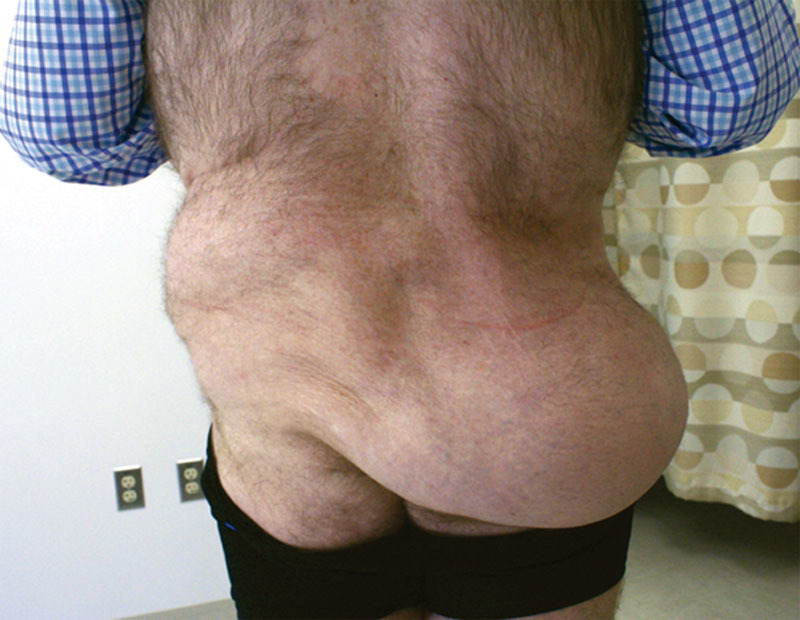
Preoperative photograph demonstrating posttraumatic pseudolipoma.

**Fig. 2.**
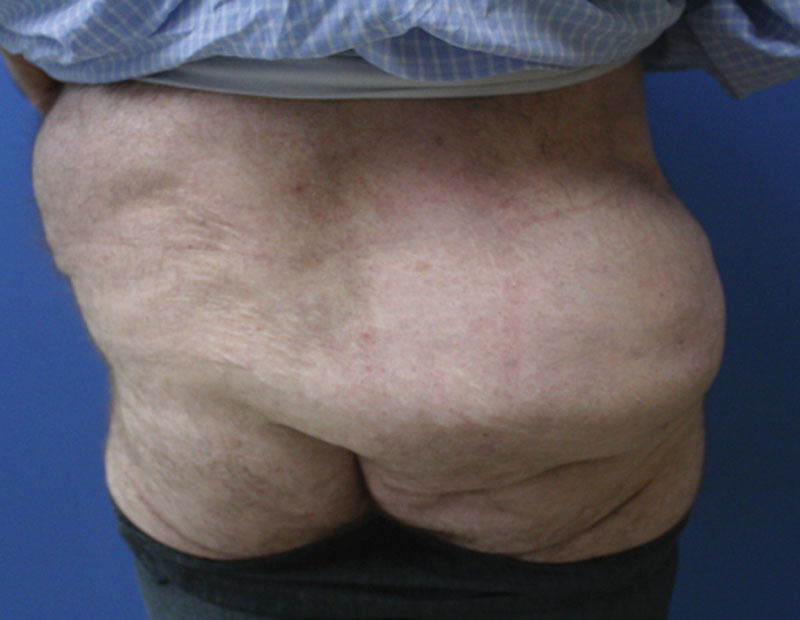
Three months postoperative photograph after debulking via suction-assisted lipectomy.

A 44-year-old male presented to our clinic with a massive soft-tissue mass that had manifested in the superior buttock immediately after falling from a bicycle 3 months prior (Fig. 1). The patient had concomitant traumatic injuries of the spine, and the buttock had been dismissed by his early treating physicians as hematoma and soft-tissue swelling. The mass failed to subside in size, interfered with routine tasks, and made wearing normal clothes challenging. It was accompanied by a notable concavity of the ipsilateral “love handle” region. A computed tomography scan showed a 30 cm accumulation of adipose tissue in the right buttock, consistent with translocation of fat from the lateral flank to the superior buttock. The patient underwent debulking of the site via suction-assisted lipectomy with significant improvement in contour and ability to accomplish daily functions at 3 months postoperatively (Fig. 2).

Normal adipose tissue is comprised of fat lobules contained within supportive fascial layers. The fascia prevents displacement of fat, but is elastic enough to allow for temporary alteration in shape to cushion deep structures when moderate external forces are applied. However, upon exposure to excessive mechanical force, the fascia is injured, and deep fat can then herniate through the fascial defects. This herniated tissue forms accumulations of normal fatty tissue from deep fat deposits in aberrant heterotopic locations.^1–5^ The majority of previous reports describe a high-energy traumatic event leading to hematoma formation and a resulting latent period (several months to years) during which pseudolipoma formation occurs.

Aside from our patient’s male sex, the immediate initial presentation lies in contrast to previous reports of identification of the pseudolipoma months to years after trauma. Whereas prevailing hypotheses point toward rents in fascia allowing progressive herniation of fat, this case is consistent with immediate degloving of the fat from an adjacent anatomical structure, with heterotopic positioning through torn interlobular septa. In addition, the “donor” site deformity and amount of translocated tissue is unique from previous reports on a purely quantitative level. It remains our opinion that early diagnosis and management of posttraumatic fat herniation should remain in the armamentarium of any provider treating blunt trauma.
